# Minimizing Uniformly Convex Functions by Cubic Regularization of Newton Method

**DOI:** 10.1007/s10957-021-01838-7

**Published:** 2021-03-10

**Authors:** Nikita Doikov, Yurii Nesterov

**Affiliations:** 1 ICTEAM (Catholic University of Louvain), Louvain-la-Neuve, Belgium; 2 CORE (Catholic University of Louvain), Louvain-la-Neuve, Belgium

**Keywords:** Newton method, Cubic regularization, Global complexity bounds, Strong convexity, Uniform convexity, 49M15, 49M37, 58C15, 90C25, 90C30

## Abstract

In this paper, we study the iteration complexity of cubic regularization of Newton method for solving composite minimization problems with uniformly convex objective. We introduce the notion of second-order condition number of a certain degree and justify the linear rate of convergence in a nondegenerate case for the method with an adaptive estimate of the regularization parameter. The algorithm automatically achieves the best possible global complexity bound among different problem classes of uniformly convex objective functions with Hölder continuous Hessian of the smooth part of the objective. As a byproduct of our developments, we justify an intuitively plausible result that the global iteration complexity of the Newton method is always better than that of the gradient method on the class of strongly convex functions with uniformly bounded second derivative.

## Introduction

A big step in a second-order optimization theory is related to the global complexity guarantees which were justified in [17] for the cubic regularization of the Newton method. The following results provide a good perspective for the development of this approach, discovering accelerated [14], adaptive [4, 5] and universal [10] schemes. The latter methods can automatically adjust to a smoothness properties of the particular objective function. In the same vein, the second-order algorithms for solving a system of nonlinear equations were discovered in [13], and randomized variants for solving large-scale optimization problems were proposed in  [7–9, 12, 18].

Despite to a number of nice properties, global complexity bounds of the cubically regularized Newton method for the cases of strongly convex and uniformly convex objective are not still fully investigated, as well as the notion of second-order non-degeneracy (see discussion in Sect. 5 in [14]). We are going to address this issue in the current paper.

The rest of the paper is organized as follows. Section 2 contains all necessary definitions and main properties of the classes of uniformly convex functions and twice-differentiable functions with Hölder continuous Hessian. We introduce the notion of the *condition number* $\gamma _{f}(\nu )$ of a certain degree $\nu \in [0, 1]$ and present some basic examples.

In Sect. 3, we describe a general regularized Newton scheme and show the linear rate of convergence for this method on the class of uniformly convex functions with a known degree $\nu \in [0, 1]$ of nondegeneracy. Then, we introduce the adaptive cubically regularized Newton method and collect useful inequalities and properties, which are related to this algorithm.

In Sect. 4, we study global iteration complexity of the cubically regularized Newton method on the classes of uniformly convex functions with Hölder continuous Hessian. We show that for nondegeneracy of *any* degree $\nu \in [0, 1]$, which is formalized by the condition $\gamma _{f}(\nu )> 0$, the algorithm automatically achieves the linear rate of convergence with the value $\gamma _{f}(\nu )$ being the main complexity factor.

Finally, in Sect. 5 we compare our complexity bounds with the known bounds for other methods and discuss the results. In particular, we justify an intuitively plausible (but quite a delayed) result that the global complexity of the cubically regularized Newton method is always better than that of the gradient method on the class of strongly convex functions with uniformly bounded second derivative.

## Uniformly Convex Functions with Hölder Continuous Hessian

Let us start from some notation. In what follows, we denote by ${\mathbb {E}}$ a finite-dimensional real vector space and by ${\mathbb {E}}^{*}$ its dual space, which is a space of linear functions on ${\mathbb {E}}$. The value of function $s \in {\mathbb {E}}^{*}$ at point $x \in {\mathbb {E}}$ is denoted by $\langle s, x \rangle$. Let us fix some linear self-adjoint positive-definite operator $B: {\mathbb {E}}\rightarrow {\mathbb {E}}^{*}$ and introduce the following Euclidean norms in the primal and dual spaces:

$$\begin{aligned} \begin{array}{rcl} \Vert x\Vert:= & {} \langle Bx, x \rangle ^{1/2}, \quad x \in {\mathbb {E}}, \qquad \Vert s\Vert _{*} \; :=\; \langle s, B^{-1}s \rangle ^{1/2}, \quad s \in {\mathbb {E}}^{*}. \end{array} \end{aligned}$$

For any linear operator $A: {\mathbb {E}}\rightarrow {\mathbb {E}}^{*}$, its norm is induced in a standard way:

$$\begin{aligned} \begin{array}{rcl} \Vert A\Vert:= & {} \max \limits _{x \in {\mathbb {E}}} \bigl \{ \Vert Ax\Vert _{*} \; | \; \Vert x\Vert \le 1 \bigr \}. \end{array} \end{aligned}$$

Our goal is to solve the convex optimization problem in the composite form:

1 $$\begin{aligned} \begin{array}{rcl} \min \limits _{x \in {\text {dom}}F} F(x):= & {} f(x) + h(x), \end{array} \end{aligned}$$

where *f* is a twice differentiable on its open domain uniformly convex function, and *h* is a *simple* closed convex function with ${\text {dom}}h \subseteq {\text {dom}}f$. *Simple* means that all auxiliary subproblems with an explicit presence of *h* are easily solvable.

For a smooth function *f*, its gradient at point *x* is denoted by $\nabla f(x) \in {\mathbb {E}}^{*}$, and its Hessian is denoted by $\nabla ^2 f(x) : {\mathbb {E}}\rightarrow {\mathbb {E}}^{*}$. For convex but not necessary differentiable function *h*, we denote by $\partial h(x) \subset {\mathbb {E}}^{*}$ its subdifferential at the point $x \in {\text {dom}}h$.

We say that differentiable function *f* is *uniformly convex* of degree $p \ge 2$ on a convex set $C \subseteq {\text {dom}}f$ if for some constant $\sigma > 0$ it satisfies inequality

2 $$\begin{aligned} \begin{array}{rcl} f(y)&\; \ge \;&f(x) + \langle \nabla f(x), y - x \rangle + \frac{\sigma }{p}\Vert y - x\Vert ^p, \qquad x, y \in C. \end{array} \end{aligned}$$

Uniformly convex functions of degree $p = 2$ are known as *strongly convex*. If inequality (2) holds with $\sigma = 0$, the function *f* is called just *convex*. The following convenient condition is sufficient for function *f* to be uniformly convex on a convex set $C \subseteq {\text {dom}}f$:

### Lemma 2.1

Lemma 1 in [14]) Let for some $\sigma > 0$ and $p \ge 2$ the following inequality holds:

3 $$\begin{aligned} \begin{array}{rcl} \langle \nabla f(x) - \nabla f(y), x - y \rangle&\; \ge \;&\sigma \Vert x - y\Vert ^p, \qquad x, y \in C. \end{array} \end{aligned}$$

Then, function *f* is uniformly convex of degree *p* on set *C* with parameter $\sigma$.

From now on, we assume $C \; := \; {\text {dom}}F \; \subseteq \; {\text {dom}}f.$ By the composite representation (1), we have for every $x \in {\text {dom}}F$ and for all $F'(x) \in \partial F(x)$:

4 $$\begin{aligned} \begin{array}{rcl} F(y)\ge & {} F(x) + \langle F'(x), y - x \rangle + \frac{\sigma }{p}\Vert x - y\Vert ^p, \qquad y \in {\text {dom}}F. \end{array} \end{aligned}$$

Therefore, if $\sigma > 0$, then we can have only one point $x^{*} \in {\text {dom}}F$ with $F(x^{*}) = F^{*}$, which always exists for *F* being uniformly convex and closed. A useful consequence of uniform convexity is the following upper bound for the residual.

### Lemma 2.2

Let *f* be uniformly convex of degree $p \ge 2$ with constant $\sigma > 0$ on set ${\text {dom}}F$. Then, for every $x \in {\text {dom}}F$ and for all $F'(x) \in \partial F(x)$ we have

5 $$\begin{aligned} \begin{array}{rcl} F(x) - F^{*}&\; \le \;&\frac{p - 1}{p} \left( \frac{1}{\sigma } \right) ^{\frac{1}{p - 1}} \Vert F'(x)\Vert _{*}^{\frac{p}{p - 1}}. \end{array} \end{aligned}$$

### Proof

In view of (4), bound (5) follows as in the proof of Lemma 3 in [14]. $\square$

It is reasonable to define the best possible constant $\sigma$ in inequality (3) for a certain degree *p*. This leads us to a system of constants:

6 $$\begin{aligned} \begin{array}{rcl} \sigma _{\!f}(p)&{} \; :=\; &{} \inf \limits _{\begin{array}{c} x, y \, \in \, {\text {dom}}F \\ x \not = y \end{array}} \frac{\langle \nabla f(x) - \nabla f(y), x - y \rangle }{\Vert x - y\Vert ^p}, \qquad p \ge 2. \end{array} \end{aligned}$$

We prefer to use inequality (3) for the definition of $\sigma _{\!f}(p)$, instead of (2), because of its symmetry in *x* and *y*. Note that the value $\sigma _{\!f}(p)$ also depends on the domain of *F*. However, we omit this dependence in our notation since it is always clear from the context.

It is easy to see that the univariate function $\sigma _{f}(\cdot )$ is log-concave. Thus, for all $p_2 > p_1 \ge 2$ we have:

7 $$\begin{aligned} \begin{array}{rcl} \sigma _{f}(p)&\; \ge \;&\bigl ( \sigma _{f}(p_1) \bigr )^{\frac{p_2 - p}{p_2 - p_1}} \cdot \bigl ( \sigma _{f}(p_2) \bigr )^{\frac{p - p_1}{p_2 - p_1}}, \qquad p \in [p_1, p_2]. \end{array} \end{aligned}$$

For a twice-differentiable function *f*, we say that it has *Hölder continuous Hessian* of degree $\nu \in [0, 1]$ on a convex set $C \subseteq {\text {dom}}f$, if for some constant ${\mathcal {H}}$, it holds:

8 $$\begin{aligned} \begin{array}{rcl} \Vert \nabla ^2 f(x) - \nabla ^2 f(y) \Vert\le & {} {\mathcal {H}} \Vert x - y\Vert ^{\nu }, \qquad x, y \in C. \end{array} \end{aligned}$$

Two simple consequences of (8) are as follows:

9 $$\begin{aligned}&\Vert \nabla f(y) - \nabla f(x) - \nabla ^2 f(x)(y - x) \Vert _{*} \le \frac{{\mathcal {H}}\Vert x - y\Vert ^{1 + \nu }}{1 + \nu }, \end{aligned}$$

10 $$\begin{aligned}&| f(y) - Q(x; y) | \le \frac{{\mathcal {H}}\Vert x - y\Vert ^{2 + \nu }}{(1 + \nu )(2 + \nu )}, \end{aligned}$$

where *Q*(*x*; *y*) is the quadratic model of *f* at the point *x*:

$$\begin{aligned} \begin{array}{rcl} Q(x; y)&\; :=\;&f(x) + \langle \nabla f(x), y - x \rangle + \frac{1}{2} \langle \nabla ^2 f(x) (y - x), y - x \rangle . \end{array} \end{aligned}$$

In order to characterize the level of smoothness of function *f* on the set $C := {\text {dom}}F$, let us define the system of Hölder constants (see [10]):

11 $$\begin{aligned} \begin{array}{rcl} {\mathcal {H}}_{\!f}(\nu )&{} \; :=&{}\; \sup \limits _{\begin{array}{c} x, y \in {\text {dom}}F \\ x \not = y \end{array}} \frac{\Vert \nabla ^2 f(x) - \nabla ^2 f(y) \Vert }{\Vert x - y\Vert ^{\nu }}, \qquad \nu \in [0, 1]. \end{array} \end{aligned}$$

We allow ${\mathcal {H}}_{\!f}(\nu )$ to be equal to $+\infty$ for some $\nu$. Note that function ${\mathcal {H}}_{f}( \cdot )$ is log-convex. Thus, any $0 \le \nu _1 < \nu _2 \le 1$ such that ${\mathcal {H}}_{f}(\nu _i) < +\infty , i = 1,2$, provide us with the following upper bounds for the whole interval:

12 $$\begin{aligned} \begin{array}{rcl} {\mathcal {H}}_{f}(\nu )&\; \le \;&\bigl ( {\mathcal {H}}_{f}(\nu _1) \bigr )^{\frac{\nu _2 - \nu }{\nu _2 - \nu _1}} \cdot \bigl ( {\mathcal {H}}_{f}(\nu _2) \bigr )^{\frac{\nu - \nu _1}{\nu _2 - \nu _1}}, \qquad \nu \in [\nu _1, \nu _2]. \end{array} \end{aligned}$$

If for some specific $\nu \in [0, 1]$ we have ${\mathcal {H}}_{\!f}(\nu )= 0$, this implies that $\nabla ^2 f(x) = \nabla ^2 f(y)$ for all $x, y \in {\text {dom}}F$. In this case restriction, $\left. f\right| _{{\text {dom}}F}$ is a quadratic function and we conclude that ${\mathcal {H}}_{\!f}(\nu )= 0$ for *all* $\nu \in [0, 1]$. At the same time, having two points $x, y \in {\text {dom}}F$ with $0 < \Vert x - y\Vert \le 1$, we get a simple uniform lower bound for all constants ${\mathcal {H}}_{\!f}(\nu )$:

$$\begin{aligned} \begin{array}{rcl} {\mathcal {H}}_{\!f}(\nu )&\; \ge \;&\Vert \nabla ^2 f(x) - \nabla ^2 f(y) \Vert , \qquad \nu \in [0, 1]. \end{array} \end{aligned}$$

Let us give an example of function, which has Hölder continuous Hessian for all $\nu \in [0, 1]$.

### Example 2.1

For a given $a_i \in {\mathbb {E}}^{*}$, $1 \le i \le m$, consider the following convex function:

$$\begin{aligned} \begin{array}{rcl} f(x)&\; = \;&\ln \left( \sum \limits _{i = 1}^m e^{\langle a_i, x \rangle } \right) , \quad x \in {\mathbb {E}}. \end{array} \end{aligned}$$

Let us fix Euclidean norm $\Vert x\Vert = \langle Bx, x \rangle ^{1/2}, x \in {\mathbb {E}}$, with operator $B := \sum _{i = 1}^m a_i a_i^{*}$. Without loss of generality, we assume that $B \succ 0$ (otherwise we can reduce dimension of the problem). Then,

$$\begin{aligned} \begin{array}{rcl} {\mathcal {H}}_{\!f}(0)&\; \le \;&1, \quad {\mathcal {H}}_{\!f}(1) \;\, \le \,\; 2. \end{array} \end{aligned}$$

Therefore, by (12) we get, for any $\nu \in [0, 1]$:

$$\begin{aligned} \begin{array}{rcl} {\mathcal {H}}_{\!f}(\nu )&\; \le \;&2^{\nu }. \end{array} \end{aligned}$$

### Proof

Denote $\kappa (x) \equiv \sum _{i = 1}^m e^{\langle a_i, x \rangle }$. Let us fix arbitrary $x, y \in {\mathbb {E}}$ and direction $h \in {\mathbb {E}}$. Then, straightforward computation gives:

$$\begin{aligned} \langle \nabla f(x), h \rangle= & {} \frac{1}{\kappa (x)} \sum _{i = 1}^m e^{\langle a_i, x \rangle } \langle a_i, h \rangle , \\ \langle \nabla ^2 f(x)h, h \rangle= & {} \frac{1}{\kappa (x)} \sum _{i = 1}^m e^{\langle a_i, x \rangle } \langle a_i, h \rangle ^2 - \bigl ( \frac{1}{\kappa (x)} \sum _{i = 1}^m e^{\langle a_i, x \rangle } \langle a_i, h \rangle \bigr )^2 \\= & {} \frac{1}{\kappa (x)} \sum _{i = 1}^m e^{\langle a_i, x \rangle } \left( \langle a_i, h \rangle - \langle \nabla f(x), h \rangle \right) ^2 \; \ge \; 0. \end{aligned}$$

Hence, we get

$$\begin{aligned} \begin{array}{rcl} \Vert \nabla ^2 f(x) \Vert= & {} \max \limits _{\Vert h\Vert \le 1} \langle \nabla ^2 f(x) h, h \rangle \; \le \; \max \limits _{\Vert h\Vert \le 1} \sum _{i = 1}^m \langle a_i, h \rangle ^2 \; = \; \max \limits _{\Vert h\Vert \le 1} \Vert h\Vert ^2 \; = \; 1. \end{array} \end{aligned}$$

Since all Hessians of function *f* are positive definite, we conclude that ${\mathcal {H}}_{\!f}(0) \le 1$. Inequality ${\mathcal {H}}_{\!f}(1) \le 2$ can be easily obtained from the following representation of the third derivative:

$$\begin{aligned} f'''(x)[h,h,h]= & {} {1 \over \kappa (x)} \sum \limits _{i=1}^m e^{\langle a_i, x \rangle } \left( \langle a_i, h \rangle - \langle \nabla f(x), h \rangle \right) ^3\\\le & {} \langle \nabla ^2 f(x) h, h \rangle \max \limits _{1 \le i,j \le m } \langle a_i - a_j , h \rangle \; \le \; 2 \Vert h \Vert ^3. \end{aligned}$$

$$\square $$

Let us imagine now that we want to describe the iteration complexity of some method, which solves the composite optimization problem (1) up to an absolute accuracy $\epsilon > 0$ in the function value. We assume that the smooth part *f* of its objective is uniformly convex and has Hölder continuous Hessians. Which degrees *p* and $\nu$ should be used in our analysis? Suppose that, for the number of *calls of the oracle*, we are interested in obtaining a polynomial-time bound of the form:

$$\begin{aligned} \begin{array}{c} O\left( ({\mathcal {H}}_{\!f}(\nu ))^{\alpha } \cdot (\sigma _{\!f}(p))^{\beta } \cdot \log \frac{F(x_0) - F^{*} \,}{\varepsilon }\right) , \quad \alpha ,\beta \ne 0. \end{array} \end{aligned}$$

Denote by $[x ]$ the *physical dimension* of variable $x \in {\mathbb {E}}$, and by $[f ]$ the *physical dimension* of the value *f*(*x*). Then, we have $[\nabla f(x) ]= [f ]/ [x ]$ and $[\nabla ^2f(x) ]= [f ]/ [x ]^2$. This gives us

$$\begin{aligned} \begin{array}{c} [{\mathcal {H}}_{\!f}(\nu )]\; = \; \frac{ [f ]}{ [x ]^{2 + \nu } }, \quad [\sigma _{\!f}(p)]= \frac{[f ]}{[x ]^p}, \quad [\, ({\mathcal {H}}_{\!f}(\nu ))^{\alpha } \cdot (\sigma _{\!f}(p))^{\beta } \, ]= \frac{[f ]^{\alpha + \beta }}{ [x ]^{\alpha (2 + \nu ) + \beta p} }. \end{array} \end{aligned}$$

While *x* and *f*(*x*) can be measured in arbitrary physical quantities, the value “number of iterations” *cannot have* physical dimension. This leads to the following relations:

$$\begin{aligned} \alpha + \beta = 0 \qquad \text {and} \qquad \alpha (2 + \nu ) + \beta p = 0. \end{aligned}$$

Therefore, despite to the fact that our function can belong to several problem classes simultaneously, from the physical point of view only one option is available:

$$\begin{aligned} \boxed {p = 2 + \nu } \end{aligned}$$

Hence, for a twice-differentiable convex function *f* with $\inf _{\nu \in [0, 1]} {\mathcal {H}}_{\!f}(\nu )> 0$, we can define only one meaningful *condition number* of degree $\nu \in [0, 1]$:

13 $$\begin{aligned} \begin{array}{rcl} \gamma _f(\nu ) \; :=\; \frac{\sigma _f(2 + \nu )}{{\mathcal {H}}_{\!f}(\nu )}. \end{array} \end{aligned}$$

If for some particular $\nu$ we have ${\mathcal {H}}_{\!f}(\nu )= +\infty$, then by our definition: $\gamma _f(\nu ) = 0$.

It will be shown that the condition number $\gamma _f(\nu )$ serves as a main factor in the global iteration complexity bounds for the regularized Newton method as applied to the problem (1). Let us prove that this number cannot be big.

### Lemma 2.3

Let $\inf _{\nu \in [0, 1]} {\mathcal {H}}_{\!f}(\nu )> 0$ and therefore the condition number $\gamma _f(\cdot )$ be well defined. Then,

14 $$\begin{aligned} \begin{array}{rcl} \gamma _f(\nu )&\quad \le \quad&\frac{1}{1 + \nu } \;\; + \; \inf \limits _{x, y \in {\text {dom}}F} \frac{\Vert \nabla ^2 f(x) \Vert }{\Vert \nabla ^2 f(y) - \nabla ^2 f(x) \Vert }, \qquad \nu \in [0, 1]. \end{array} \end{aligned}$$

In the case when ${\text {dom}}F$ is unbounded: $\sup _{x \in {\text {dom}}F} \Vert x\Vert = +\infty$, then

15 $$\begin{aligned} \begin{array}{rcl} \gamma _f(\nu )&\quad \le \quad&\frac{1}{1 + \nu }, \qquad \nu \in (0, 1]. \end{array} \end{aligned}$$

### Proof

Indeed, for any $x, y \in {\text {dom}}F$, $x \not = y$, we have:

$$\begin{aligned}&\sigma _f(2 + \nu ) \quad \overset{(6)}{\le } \quad \frac{\langle \nabla f(y) - \nabla f(x), y - x \rangle }{\Vert y - x\Vert ^{2 + \nu }} \\&\quad = \quad \frac{\langle \nabla f(y) - \nabla f(x) - \nabla ^2 f(x)(y - x), y - x \rangle }{\Vert y - x\Vert ^{2 + \nu }} \; + \; \frac{\langle \nabla ^2 f(x)(y - x), y - x \rangle }{\Vert y - x\Vert ^{2 + \nu }} \\&\quad \overset{(9)}{\le } \quad \frac{{\mathcal {H}}_{\!f}(\nu )}{1 + \nu } \; + \; \frac{\Vert \nabla ^2 f(x) \Vert }{\Vert y - x\Vert ^{\nu }}. \end{aligned}$$

Now, dividing both sides of this inequality by ${\mathcal {H}}_{\!f}(\nu )$, we get inequality (14) from the definition of ${\mathcal {H}}_{\!f}(\nu )$ (11). Inequality (15) can be obtained by taking the limit $\Vert y\Vert \rightarrow +\infty$. $\square$

From inequalities (7) and (12), we can get the following lower bound:

$$\begin{aligned} \begin{array}{rcl} \gamma _f(\nu )&\; \ge \;&\bigl ( \gamma _f(\nu _1) \bigr )^{\frac{\nu _2 - \nu }{\nu _2 - \nu _1}} \cdot \bigl ( \gamma _f(\nu _2) \bigr )^{\frac{\nu - \nu _1}{\nu _2 - \nu _1}}, \qquad \nu \in [\nu _1, \nu _2], \end{array} \end{aligned}$$

where $0 \le \nu _1 < \nu _2 \le 1$. However, it turns out that in *unbounded case* we can have a nonzero condition number $\gamma _{f}(\nu )$ only for a *single degree*.

### Lemma 2.4

  Let ${\text {dom}}F$ be unbounded: $\sup _{x \in {\text {dom}}F} \Vert x\Vert = +\infty$. Assume that for a fixed $\nu \in [0, 1]$ we have $\gamma _f(\nu ) > 0$. Then,

$$\begin{aligned} \gamma _f(\alpha ) = 0 \quad \text {for all} \quad \alpha \in [0, 1] \setminus \{ \nu \}. \end{aligned}$$

### Proof

Consider firstly the case: $\alpha > \nu$. From the condition $\gamma _f(\nu ) > 0$, we conclude that ${\mathcal {H}}_{\!f}(\nu ) < +\infty$. Then, for any $x, y \in {\text {dom}}F$ we have:

$$\begin{aligned}&\frac{\sigma _{\!f}(2 + \alpha ) \Vert y - x\Vert ^{2 + \alpha }}{2 + \alpha } \quad \overset{(2)}{\le } \quad f(y) - f(x) - \langle \nabla f(x), y - x \rangle \\&\quad \overset{(10)}{\le } \quad \frac{1}{2}\langle \nabla ^2 f(x)(y - x), (y - x) \rangle + \frac{{\mathcal {H}}_{\!f}(\nu ) \Vert y - x\Vert ^{2 + \nu }}{(1 + \nu )(2 + \nu )}. \end{aligned}$$

Dividing both sides of this inequality by $\Vert y - x\Vert ^{2 + \alpha }$ and letting $\Vert x\Vert \rightarrow +\infty$, we get $\sigma _{\!f}(2 + \nu ) = 0$. Therefore, $\gamma _f(\alpha ) = 0$. For the second case, $\alpha < \nu$, we cannot have $\gamma _f(\alpha ) > 0$, since the previous reasoning results in $\gamma _f(\nu ) = 0$. $\square$

Let us look now at an important example of a uniformly convex function with Hölder continuous Hessian. It is convenient to start with some properties of powers of Euclidean norm.

### Lemma 2.5

For fixed real $p\ge 1$, consider the following function:

$$\begin{aligned} \begin{array}{rcl} f_p(x) \; = \; \frac{1}{p} \Vert x \Vert ^{p}, \quad x \in {\mathbb {E}}. \end{array} \end{aligned}$$

1. For $$p \ge 2$$, function $$f_p(\cdot )$$ is uniformly convex of degree *p*:^1^$$^{)}$$

16 $$\begin{aligned} \langle \nabla f_p(x) - \nabla f_p(y), x - y \rangle \quad \ge \quad 2^{2 - p} \Vert x - y\Vert ^{p}, \quad x, y \in {\mathbb {E}}. \end{aligned}$$

2. If $1 \le p \le 2$, then function $f_p(\cdot )$ has $\nu$-Hölder continuous gradient with $\nu = p-1$:

17 $$\begin{aligned} \Vert \nabla f_p(x) - \nabla f_p(y) \Vert _* \le 2^{1-\nu } \Vert x - y \Vert ^{\nu }, \quad x, y \in {\mathbb {E}}. \end{aligned}$$

### Proof

Firstly, recall two useful inequalities, which are valid for all $a, b \ge 0$:

18 $$\begin{aligned}&|a^{\alpha } - b^{\alpha }| \; \le \; |a - b|^{\alpha }, \quad \text {when} \quad 0 \le \alpha \le 1, \end{aligned}$$

19 $$\begin{aligned}&|a^{\alpha } - b^{\alpha }| \; \ge \; |a - b|^{\alpha }, \quad \text {when} \quad \alpha \ge 1. \end{aligned}$$

Let us fix arbitrary $x, y \in {\mathbb {E}}$. The left-hand side of inequality (16) equals

$$\begin{aligned} \langle \Vert x\Vert ^{p - 2}Bx - \Vert y\Vert ^{p - 2}By, x - y \rangle \; = \; \Vert x\Vert ^p + \Vert y\Vert ^p - \langle Bx, y \rangle ( \Vert x\Vert ^{p - 2} + \Vert y\Vert ^{p - 2} ), \end{aligned}$$

and we need to verify that it is bigger than $2^{2 - p}\bigl [ \Vert x\Vert ^2 + \Vert y\Vert ^2 - 2 \langle Bx, y \rangle \bigr ]^{\frac{p}{2}}.$ The case $x = 0$ or $y = 0$ is trivial. Therefore, assume $x \not = 0$ and $y \not = 0$. Denoting $\tau := \frac{\Vert y\Vert }{\Vert x\Vert }$, $r := \frac{\langle Bx, y\rangle }{\Vert x\Vert \cdot \Vert y\Vert }$, we have the following statement to prove:

$$\begin{aligned} \begin{array}{rcl} 1 + \tau ^p\ge & {} r \tau (1 + \tau ^{p - 2}) + 2^{2 - p} \bigl [ 1 + \tau ^2 - 2 r \tau \bigr ]^{\frac{p}{2}} , \quad \tau > 0, \quad |r| \le 1. \end{array} \end{aligned}$$

Since the function in the right-hand side is convex in *r*, we need to check only two marginal cases:

1. $r = 1 \, : \quad$ $1 + \tau ^{p} \; \ge \; \tau (1 + \tau ^{p - 2}) + 2^{2 - p} |1 - \tau |^p$, which is equivalent to $(1 - \tau ) (1 - \tau ^{p - 1}) \ge 2^{2 - p}|1 - \tau |^p$. This is true by (19).
2. $r = -1\, : \quad$ $1 + \tau ^{p} \; \ge \; -\tau (1 + \tau ^{p - 2}) + 2^{2 - p}(1 + \tau )^p$, which is equivalent to $(1 + \tau ^{p - 1}) \ge 2^{2 - p}(1 + \tau )^{p - 1}$. This is true in view of convexity of function $\tau ^{p-1}$ for $\tau \ge 0$.

Thus, we have proved (16). Let us prove the second statement. Consider the function ${\hat{f}}_q(s) = {1 \over q} \Vert s \Vert ^q_*$, $s \in {\mathbb {E}}^*$, with $q = {p \over p-1} \ge 2$. In view of our first statement, we have:

20 $$\begin{aligned} \begin{array}{rcl} \langle s_1 - s_2, \nabla {\hat{f}}_q(s_1) - \nabla {\hat{f}}_q(s_2) \rangle\ge & {} \left( {1 \over 2}\right) ^{q-2} \Vert s_1 - s_2 \Vert _*^q, \quad s_1, s_2 \in {\mathbb {E}}^*. \end{array} \end{aligned}$$

For arbitrary $x_1, x_2 \in {\mathbb {E}}$, define $s_i = \nabla f_p(x_i) = {B x_i \over \Vert x_i \Vert ^{2-p}}$, $i = 1, 2$. Then $\Vert s_i \Vert _* = \Vert x_i \Vert ^{p-1}$, and consequently,

$$\begin{aligned} \begin{array}{rcl} x_i= & {} \Vert x_i \Vert ^{2-p} B^{-1} s_i \; = \; \Vert s_i \Vert _{*}^{2-p \over p-1} B^{-1} s_i \; = \; \nabla {\hat{f}}_q(s_i). \end{array} \end{aligned}$$

Therefore, substituting these vectors in (20), we get

$$\begin{aligned} \left( {1 \over 2}\right) ^{q-2} \Vert \nabla f_p(x_1) - \nabla f_p(x_2) \Vert _*^q \le \langle \nabla f_p(x_1) - \nabla f_p(x_2), x_1 - x_2 \rangle . \end{aligned}$$

Thus, $\Vert \nabla f_p(x_1) - \nabla f_p(x_2) \Vert _* \le 2^{q-2 \over q-1} \Vert x_1 - x_2 \Vert ^{1 \over q-1}$. It remains to note that ${1 \over q-1} = p-1 = \nu$. $\square$

### Example 2.2

For real $p \ge 2$ and arbitrary $x_0 \in {\mathbb {E}}$, consider the following function:

$$\begin{aligned} \begin{array}{rcl} f(x) \; = \; \frac{1}{p} \Vert x - x_0\Vert ^{p} \; = \; f_p(x - x_0), \quad x \in {\mathbb {E}}. \end{array} \end{aligned}$$

Then, $\sigma _{\!f}(p) \; = \; \left( \frac{1}{2} \right) ^{p - 2}$. Moreover, if $p = 2 + \nu$ for some $\nu \in (0, 1]$, then it holds

$$\begin{aligned} \begin{array}{rcl} {\mathcal {H}}_{\!f}(\nu )\; \le \; (1 + \nu )2^{1 - \nu }, \end{array} \end{aligned}$$

and ${\mathcal {H}}_{\!f}(\alpha ) \; = \; +\infty$, for all $\alpha \in [0, 1] \setminus \{\nu \}$. Therefore, in this case we have $\gamma _{f}(\nu )\; \ge \; \frac{1}{2(1 + \nu )},$ and $\gamma _{f}(\alpha ) = 0$ for all $\alpha \in [0, 1] \setminus \{\nu \}$.

### Proof

Let us take an arbitrary $x \ne 0$ and set $y := -x$. Then,

$$\begin{aligned} \langle \nabla f(x) - \nabla f(y), y - x \rangle \; = \; \langle \Vert x\Vert ^{p - 2} Bx + \Vert x\Vert ^{p - 2} Bx, 2 x \rangle \; = \; 4 \Vert x\Vert ^{p}. \end{aligned}$$

On the other hand, $\Vert y - x \Vert ^p = 2^p \Vert x \Vert ^p$. Therefore, $\sigma _{\!f}(p) {\mathop {\le }\limits ^{(6)}} 2^{2-p}$, and (16) tells us that this inequality is satisfied as equality.

Let us prove now that ${\mathcal {H}}_{\!f}(\nu )\le (1 + \nu )2^{1 - \nu }$ for $p = 2 + \nu$ with some $\nu \in (0, 1]$. This is

21 $$\begin{aligned} \Vert \nabla ^2 f(x) - \nabla ^2 f(y) \Vert \; \le \; (1 + \nu ) 2^{1 - \nu } \Vert x - y\Vert ^{\nu }, \quad x, y \in {\mathbb {E}}. \end{aligned}$$

The corresponding Hessians can be represented as follows:

$$\begin{aligned} \begin{array}{rcl} \nabla ^2 f(x)= & {} \Vert x\Vert ^{\nu } B + \frac{\nu B x x^{*} B}{\Vert x\Vert ^{2 - \nu }}, \quad x \in {\mathbb {E}}\setminus \{0\}, \qquad \nabla ^2 f(0) = 0. \end{array} \end{aligned}$$

For the case $x = y = 0$, inequality (21) is trivial. Assume now that $x \not = 0$. If $0 \in [x, y]$, then $y = -\beta x$ for some $\beta \ge 0$ and we have:

$$\begin{aligned} \Vert \nabla ^2 f(x) - \nabla ^2 f(-\beta x) \Vert\le & {} |1 - \beta ^\nu | (1 + \nu ) \Vert x\Vert ^{\nu } \; \le \; (1 + \beta )^{\nu } (1 + \nu ) 2^{1 - \nu } \Vert x\Vert ^{\nu } \\= & {} (1 + \nu ) 2^{1 - \nu } \Vert x - y\Vert ^{\nu }, \end{aligned}$$

which is (21). Let $0 \notin [x, y]$. For an arbitrary fixed direction $h \in {\mathbb {E}}$, we get:

$$\begin{aligned} \begin{array}{rcl} \bigl | \bigl \langle (\nabla ^2 f(x) - \nabla ^2 f(y)) h, h \bigr \rangle \bigr |= & {} \Bigl | \left( \Vert x\Vert ^{\nu } - \Vert y\Vert ^{\nu } \right) \cdot \Vert h\Vert ^2 + \nu \cdot \left( \frac{\langle Bx, h \rangle ^2}{\Vert x\Vert ^{2 - \nu }} - \frac{\langle By, h \rangle ^2}{\Vert y\Vert ^{2 - \nu }} \right) \Bigr |. \end{array} \end{aligned}$$

Consider the points $u = \frac{Bx}{\Vert x\Vert ^{1 - \nu }} = \nabla f_q(x)$ and $v = \frac{By}{\Vert y\Vert ^{1 - \nu }} = \nabla f_q(y)$ with $q = 1+\nu$. Then,

$$\begin{aligned} \begin{array}{c} \Vert x\Vert ^{\nu } = \Vert u\Vert _*, \quad \frac{\langle B x, h \rangle ^2}{\Vert x\Vert ^{2 - \nu }} = \frac{\langle u, h \rangle ^2}{\Vert u\Vert _*} \quad \text {and} \quad \Vert y\Vert ^{\nu } = \Vert v\Vert _*, \quad \frac{\langle By, h \rangle ^2}{\Vert y\Vert ^{2 - \nu }} = \frac{\langle v, h \rangle ^2}{\Vert v \Vert _*}. \end{array} \end{aligned}$$

Therefore,

22 $$\begin{aligned}&\bigl | \bigl \langle (\nabla ^2 f(x) - \nabla ^2 f(y)) h, h \bigr \rangle \bigr | \nonumber \\&\quad = \Bigl | \left( \Vert u\Vert _* - \Vert v\Vert _* \right) \cdot \Vert h\Vert ^2 \; + \; \nu \cdot \left( \frac{\langle u, h \rangle ^2}{\Vert u\Vert _*} - \frac{\langle v, h \rangle ^2}{\Vert v\Vert _*} \right) \Bigr |. \end{aligned}$$

Let us estimate the right-hand side of (22) from above. Consider a continuously differentiable univariate function:

$$\begin{aligned} \phi (\tau ):= & {} \Vert u(\tau )\Vert _* \cdot \Vert h\Vert ^2 + \nu \cdot \frac{\langle u(\tau ), h \rangle ^2}{\Vert u(\tau )\Vert _*}, \\ u(\tau ):= & {} u + \tau (v - u), \quad \tau \in [0, 1]. \end{aligned}$$

Note that

$$\begin{aligned} \phi ^{\prime }(\tau )= & {} \frac{\langle u(\tau ), B^{-1}(v-u)\rangle }{\Vert u(\tau )\Vert _*} \cdot \Vert h\Vert ^2 + \frac{2 \nu \langle u(\tau ), h \rangle \langle v-u, h \rangle }{\Vert u(\tau )\Vert _*} \;\\&- \; \frac{\nu \langle u(\tau ), h \rangle ^2 \langle u(\tau ), B^{-1}(v-u) \rangle }{\Vert u(\tau )\Vert _*^3}\\= & {} \frac{\langle u(\tau ), B^{-1}(v-u) \rangle }{\Vert u(\tau )\Vert _*} \cdot \underbrace{\left( \Vert h\Vert ^2 - \tfrac{\nu \langle u(\tau ), h \rangle ^2}{\Vert u(\tau )\Vert _*^2} \right) }_{\ge 0} \; + \; \frac{2\nu \langle u(\tau ), h \rangle \langle v-u, h\rangle }{\Vert u(\tau ) \Vert _*}. \end{aligned}$$

Denote $$\gamma := \frac{\langle u(\tau ), h \rangle }{\Vert u(\tau )\Vert _* \cdot \Vert h\Vert } \in [-1, 1]$$. Then,

$$\begin{aligned} \bigl | \phi ^{\prime }(\tau ) \bigr | \; \le \; \Vert v - u\Vert _* \cdot \Vert h\Vert ^2 \cdot \bigl (1 - \nu \gamma ^2 + 2\nu |\gamma | \bigr ) \; \le \; (1 + \nu ) \cdot \Vert v-u\Vert _* \cdot \Vert h\Vert ^2. \end{aligned}$$

Thus, we have:

23 $$\begin{aligned} \bigl | \bigl \langle (\nabla ^2 f(x) - \nabla ^2 f(y)) h, h \bigr \rangle \bigr | \; = \; | \phi (1) - \phi (0) | \; \le \; (1 + \nu ) \cdot \Vert v - u\Vert _* \cdot \Vert h\Vert ^2. \end{aligned}$$

It remains to use the definition of *u* and *v* and apply inequality (17) with $p=q$. Thus, we have proved, that for $p = 2 + \nu$ the Hessian of *f* is Hölder continuous of degree $\nu$. At the same time, taking $y = 0$, we get $\Vert \nabla ^2 f(x) - \nabla ^2 f(y) \Vert = \Vert \nabla ^2 f(x) \Vert = (1 + \nu )\Vert x\Vert ^{\nu }$. These values cannot be uniformly bounded in $x \in {\mathbb {E}}$ by any multiple of $\Vert x\Vert ^{\alpha }$ with $\alpha \ne \nu$. So, the Hessian of *f* is *not* Hölder continuous for any degree different from $2+\nu$. $\square$

### Remark 2.1

Inequalities (16) and (17) have the following symmetric consequences:

$$\begin{aligned} p \ge 2\Rightarrow & {} \Vert \nabla f_p(x) - \nabla f_p(y) \Vert _* \; \ge \; 2^{2-p} \Vert x - y \Vert ^{p-1}, \\ p \le 2\Rightarrow & {} \Vert \nabla f_p(x) - \nabla f_p(y) \Vert _* \; \le \; 2^{2-p} \Vert x - y \Vert ^{p-1}, \end{aligned}$$

which are valid for all $x, y \in {\mathbb {E}}$.

## Regularized Newton Method

Let us start from the case when we know that for a specific $\nu \in [0, 1]$ function *f* has Hölder continuous Hessian: ${\mathcal {H}}_f(\nu ) < +\infty$. Then, from (10), we have the global upper bound for the objective function:

$$\begin{aligned} \begin{array}{rcl} F(y)&\; \le \;&M_{\nu , H}(x; y) \;:=\; Q(x; y) + \frac{H \Vert x - y\Vert ^{2 + \nu }}{(1 + \nu )(2 + \nu )} + h(y), \qquad x, y \in {\text {dom}}F, \end{array} \end{aligned}$$

where $H > 0$ is large enough: $H \ge {\mathcal {H}}_{\!f}(\nu )$. Thus, it is natural to employ the minimum of a regularized quadratic model:

$$\begin{aligned} \begin{array}{c} T_{\nu , H}(x) \; :=\; \mathop {\mathrm{argmin}}\limits _{y \in {\text {dom}}F} M_{\nu , H}(x; y), \qquad M_{\nu , H}^{*}(x) \; :=\; \min \limits _{y \in {\text {dom}}F} M_{\nu , H}(x; y), \end{array} \end{aligned}$$

and define the following general iteration process [10]:

24 $$\begin{aligned} \boxed { \quad x_{k + 1} \; := \; T_{\nu , H_k}(x_k), \qquad k \ge 0} \end{aligned}$$

where the value $H_k$ is chosen either to be a constant from the interval $[0, 2{\mathcal {H}}_{\!f}(\nu )]$ or by some adaptive procedure.

For the class of uniformly convex functions of degree $p = 2 + \nu$, we can justify the following global convergence result for this process.

### Theorem 3.1

Assume that for some $\nu \in [0, 1]$ we have $0< {\mathcal {H}}_{\!f}(\nu )< +\infty$ and $\sigma _{\!f}(2 + \nu )> 0$. Let the coefficients $\{ H_k \}_{k \ge 0}$ in the process (24) satisfy the following conditions:

25 $$\begin{aligned} 0 \le H_k \le \beta {\mathcal {H}}_f(\nu ), \qquad F(x_{k + 1}) \le M_{\nu , H_k}^{*}(x_k), \qquad k \ge 0, \end{aligned}$$

with some constant $\beta \ge 0$. Then, for the sequence $\{x_k\}_{k \ge 0}$ generated by the process we have:

26 $$\begin{aligned} \begin{array}{rcl} F(x_{k + 1}) - F^{*}\le & {} \Bigl ( 1 \; - \; \frac{1 + \nu }{2 + \nu } \cdot \min \Bigl \{\frac{\gamma _{f}(\nu )(1 + \nu )}{(1 + \beta ) (2 + \nu )}, \, 1 \Bigr \}^{\frac{1}{1 + \nu }} \Bigr ) \left( F(x_k) - F^{*} \right) . \end{array} \end{aligned}$$

Thus, the rate of convergence is linear and for reaching the gap $F(x_K) - F^{*} \le \varepsilon$ it is enough to perform $K \; = \; \bigl \lceil \frac{2 + \nu }{1 + \nu } \cdot \max \bigl \{ \frac{(1 + \beta )(2 + \nu )}{\gamma _{f}(\nu )(1 + \nu )}, \, 1 \bigr \}^{\frac{1}{1 + \nu }} \log \frac{F(x_0) - F^{*}}{\varepsilon } \bigr \rceil$ iterations.

### Proof

As in the proof of Theorem 3.1 in [10], from (25) one can see that

$$\begin{aligned} \begin{array}{rcl} F(x_{k + 1})\le & {} F(x_k) - \alpha \left( F(x_k) - F^{*} \right) + \alpha ^{2 + \nu } \frac{(1 + \beta ) {\mathcal {H}}_{\!f}(\nu )\Vert x_k - x^{*}\Vert ^{2 + \nu }}{(1 + \nu )(2 + \nu )}, \end{array} \end{aligned}$$

for any $\alpha \in [0, 1]$. Then, taking into account the uniform convexity (4), we get

$$\begin{aligned} \begin{array}{rcl} F(x_{k + 1})\le & {} F(x_k) - \left( \alpha - \alpha ^{2 + \nu } \frac{(1 + \beta ) {\mathcal {H}}_{\!f}(\nu )}{(1 + \nu ) \sigma _{\!f}(2 + \nu )} \right) \left( F(x_k) - F^{*} \right) . \end{array} \end{aligned}$$

The minimum of the right-hand side is attained at $\alpha ^{*} = \min \bigl \{ \frac{ \gamma _{f}(\nu )(1 + \nu )}{(2 + \nu )(1 + \beta )}, 1 \bigr \}^{\frac{1}{1 + \nu }}$. Plugging this value into the bound above, we get inequality (26). $\square$

Unfortunately, in practice it is difficult to decide on an appropriate value of $\nu \in [0, 1]$ with ${\mathcal {H}}_{\!f}(\nu )< +\infty$. Therefore, it is interesting to develop the *universal methods* which are not based on some particular parameters. Recently, it was shown [10] that one good choice for such universal scheme is the cubic regularization of the Newton Method [17]. This is actually the process (24) with the fixed parameter $\nu = 1$. For this choice, in the rest part of the paper we omit the corresponding index in the definitions of all necessary objects: $M_H(x; y) := M_{1, H}(x; y)$, $T_H(x) := T_{1, H}(x)$, and $M_H^{*}(x) := M_{1, H}^{*}(x) = M_H(x; T_H(x))$. The adaptive scheme of our method with dynamic estimation of the constant *H* is as follows.

**Table:** 

**Algorithm 1: Adaptive Cubic Regularization of Newton Method**	
**Initialization.** Choose $x_0 \in {\text {dom}}F$, $H_0 > 0$.	
**Iteration** $k \ge 0$.	
1: Find the minimal integer $i_k \ge 0 \;$ such that $F(T_{H_k 2^{i_k}}(x_k)) \le M^{*}_{H_{k} 2^{i_k}} (x_k)$.	
2: Perform the Cubic Step: $x_{k + 1} = T_{H_{k} 2^{i_k}}(x_k)$.	
3: Set $$H_{k + 1} := 2^{i_k - 1} H_k $$.	

Let us present the main properties of the composite Cubic Newton step $x \mapsto T_H(x)$. Denote

$$\begin{aligned} r_H(x) :=\Vert T_H(x) - x\Vert . \end{aligned}$$

Since point $T_H(x)$ is a minimum of strictly convex function $M_H(x;\cdot )$, it satisfies the following first-order optimality condition:

27 $$\begin{aligned}&\bigl \langle \nabla f(x) + \nabla ^2 f(x)(T_H(x) - x) + \tfrac{H r_H(x)}{2}B(T_H(x) - x), y - T_H(x) \bigr \rangle \; \nonumber \\&\quad + h(y) \; \ge \; h(T_H(x)), \qquad y \in {\text {dom}}F. \end{aligned}$$

In other words, the vector

$$\begin{aligned} \begin{array}{rcl} h'(T_H(x)):= & {} -\nabla f(x) - \nabla ^2 f(x)(T_H(x) - x) - \frac{H r_H(x)}{2}B(T_H(x) - x) \end{array} \end{aligned}$$

belongs to the subdifferential of *h*:

28 $$\begin{aligned} \begin{array}{rcl} h'(T_H(x))&\; \in \;&\partial h(T_H(x)). \end{array} \end{aligned}$$

Computation of a point $T = T_H(x)$, satisfying condition (28), requires some standard techniques of Convex Optimization and Linear Algebra (see [1, 3, 16, 17]). Arithmetical complexity of such a procedure is usually similar to that of the standard Newton step.

Plugging into (27) $y := x \in {\text {dom}}F$, we get:

29 $$\begin{aligned}&\langle \nabla f(x), x - T_H(x) \rangle \nonumber \\&\quad \ge \; \langle \nabla ^2 f(x) (T_H(x) - x), T_H(x) - x \rangle + \tfrac{H r_H^3(x)}{2} + h(T_H(x)) - h(x). \end{aligned}$$

Thus, we obtain the following bound for the minimal value $M_H^{*}(x)$ of the cubic model:

30 $$\begin{aligned}&M_H^{*}(x) \overset{(29)}{\le } f(x) - \tfrac{1}{2}\langle \nabla ^2 f(x)(T_H(x) - x), T_H(x) - x \rangle - \tfrac{H r_H^3(x)}{3} + h(x) \nonumber \\&\quad = F(x) - \tfrac{1}{2}\langle \nabla ^2 f(x)(T_H(x) - x), T_H(x) - x \rangle - \tfrac{Hr_H^3(x)}{3}. \end{aligned}$$

If for some value $\nu \in [0, 1]$ the Hessian is Hölder continuous: ${\mathcal {H}}_{\!f}(\nu )< +\infty$, then by (9) and (28) we get the following bound for the subgradient:

$$\begin{aligned} F'(T_H(x)) :=\nabla f(T_H(x)) + h'(T_H(x)) \end{aligned}$$

at the new point:

31 $$\begin{aligned}&\Vert F'(T_H(x)) \Vert _{*} \nonumber \\&\quad \le \; \Vert \nabla f(T_H(x)) - \nabla f(x) - \nabla ^2 f(x) (T_H(x) - x)\Vert _{*} + \tfrac{H r^2_H(x)}{2} \nonumber \\&\quad {\mathop {\le }\limits ^{(9)}} \tfrac{{\mathcal {H}}_{\!f}(\nu )r^{1 + \nu }_H(x)}{1 + \nu } + \tfrac{H r^2_H(x)}{2} \; = \; r^{1 + \nu }_H(x) \cdot \Bigl ( \tfrac{{\mathcal {H}}_{\!f}(\nu )}{1 + \nu } + \tfrac{Hr_H^{1 - \nu }(x)}{2} \Bigr ). \end{aligned}$$

One of the main strong point of the classical Newton’s is its local *quadratic convergence* for the class of strongly convex functions with Lipschitz continuous Hessian: $\sigma _{\!f}(2) > 0$ and $0< {\mathcal {H}}_{\!f}(1) < +\infty$ (see, for example, [15]). This property holds for the cubically regularized Newton as well [14, 17]. Indeed, ensuring $F(T_H(x)) \le M_{H}^{*}(x)$ as in Algorithm 1, and having $H \le \beta {\mathcal {H}}_{\!f}(1)$ with some $\beta \ge 0$, we get:

$$\begin{aligned}&F(T_H(x)) - F^{*} \overset{(5)}{\le } \frac{1}{2\sigma _{\!f}(2)}\Vert F'(T_H(x)) \Vert _{*}^2 \; \overset{(31)}{\le } \; \frac{(1 + \beta )^2 {\mathcal {H}}_{\!f}^2(1)}{8 \sigma _{\!f}(2)} r_H^4(x) \nonumber \\&\quad \le \frac{(1 + \beta )^2 {\mathcal {H}}_{\!f}^2(1)}{8 \sigma _{\!f}^3(2)} \langle \nabla ^2 f(x)(T_H(x) - x), T_H(x) - x \rangle ^2 \nonumber \\&\quad \overset{(30)}{\le } \frac{(1 +\beta )^2 {\mathcal {H}}_{\!f}^2(1)}{2 \sigma _{\!f}^3(2)} \left( F(x)- F^{*} \right) ^2. \end{aligned}$$

And the region of quadratic convergence is as follows:

$$\begin{aligned} {\mathcal {Q}} \; = \; \bigl \{ \, x \in {\text {dom}}F \; : \; F(x) - F^{*} \; \le \; \frac{2\sigma _{\!f}^3(2)}{(1 + \beta )^2 {\mathcal {H}}_{\!f}^2(1)} \, \bigr \}. \end{aligned}$$

After reaching it, the method starts to double the right digits of the answer at every step, and this cannot last for a long time. Therefore, from now on we are mainly interested in the *global complexity bounds* of Algorithm 1, which work for an arbitrary starting point $x_0$.

For noncomposite case, as it was shown in [10], if for some $\nu \in [0, 1]$ we have $0< {\mathcal {H}}_{\!f}(\nu )< +\infty$ and the objective is just *convex*, then Algorithm 1 with small initial parameter $H_0$ generates a solution $\hat{x}$ with $f({\hat{x}}) - f^{*} \le \varepsilon$ in $O\bigl ( \bigl (\frac{{\mathcal {H}}_{\!f}(\nu )D_0^{2 + \nu }}{\varepsilon }\bigr )^{\frac{1}{1 + \nu }} \bigr )$ iterations, where $D_0 \; := \; \max \limits _{x}\left\{ \Vert x - x^{*}\Vert \; : \; f(x) \le f(x_0) \right\}$. Thus, the method in [10] has a sublinear rate of convergence on the class of convex functions with Hölder continuous Hessian. It can *automatically adapt* to the actual level of smoothness. In what follows we show that the same algorithm achieves linear rate of convergence for the class of *uniformly convex* functions of degree $p = 2 + \nu$, namely for functions with strictly positive condition number: $\sup _{\nu \in [0, 1]} \gamma _{f}(\nu )> 0.$

In the remaining part of the paper, we usually assume that the smooth part of our objective is not *purely quadratic*. This is equivalent to the condition $\inf _{\nu \in [0, 1]} {\mathcal {H}}_{\!f}(\nu )> 0$. However, to conclude this section, let us briefly discuss the case $\min _{\nu \in [0, 1]} {\mathcal {H}}_{\!f}(\nu )= 0$. If we would know in advance that *f* is a convex quadratic function, then no regularization is needed since a single step $x \mapsto T_H(x)$ with $H := 0$ solves the problem. However, if our function is given by a black-box oracle and we do not know a priori that its smooth part is quadratic, then we can still use Algorithm 1. For this case, we prove the following simple result.

### Proposition 3.1

Let $A: {\mathbb {E}}\rightarrow {\mathbb {E}}^{*}$ be a self-adjoint positive semidefinite linear operator and $b \in {\mathbb {E}}^{*}$. Assume that $f(x) \; := \; \frac{1}{2}\langle Ax, x \rangle - \langle b, x \rangle ,$ and the minimum $x^{*} \in \mathop {\mathrm{Argmin}}\limits _{x \in {\text {dom}}F} \bigl \{ F(x) := f(x) + h(x)\bigr \}$ does exist. Then, in order to get $F(x_K) - F^{*} \le \varepsilon$ with arbitrary $\varepsilon > 0$, it is enough to perform

32 $$\begin{aligned} \begin{array}{rcl} K \; = \; \bigl \lceil \log _2 \frac{H_0 \Vert x_0 - x^{*}\Vert ^3}{6 \varepsilon } \; + \; 1 \bigr \rceil \end{array} \end{aligned}$$

iterations of Algorithm 1.

### Proof

In our case, the quadratic model coincides with the smooth part of the objective: $Q(x; y) \equiv f(y), \; x, y \in {\mathbb {E}}.$ Therefore, at every iteration $k \ge 0$ of Algorithm 1 we have $i_k = 0$ and $H_k = 2^{-k} H_0$. Note that $x_{k + 1} = T_{2^{-k} H_0}(x_k) = \mathop {\mathrm{argmin}}\limits _{y \in {\text {dom}}F}\bigl \{ F(y) + \tfrac{2^{-k}H_0}{6}\Vert y - x_k\Vert ^3 \bigr \}$, and

33 $$\begin{aligned} \begin{array}{rcl} F(x_{k + 1})&\; \le \;&F(y) + \frac{2^{-k} H_0}{6}\Vert y - x_k\Vert ^3, \qquad y \in {\text {dom}}F. \end{array} \end{aligned}$$

Let us prove that $\Vert x_{k + 1} - x^{*}\Vert \le \Vert x_k - x^{*}\Vert$ for all $k \ge 0$. If this is true, then plugging $y \equiv x^{*}$ into (33), we get: $F(x_{k + 1}) - F^{*} \le 2^{-k}\frac{H_0}{6}\Vert x_0 - x^{*}\Vert ^3$ which results in the estimate (32). Indeed,

$$\begin{aligned} \Vert x_k - x^{*}\Vert ^2= & {} \Vert (x_k - x_{k + 1}) + (x_{k + 1} - x^{*}) \Vert ^2 \\= & {} \Vert x_{k + 1} - x^{*}\Vert ^2 + \Vert x_{k} - x_{k + 1} \Vert ^2 + 2\langle B(x_k - x_{k + 1}), x_{k + 1} - x^{*} \rangle , \end{aligned}$$

and it is enough to show that $\langle B(x_k - x_{k + 1}), x^{*} - x_{k + 1} \rangle \; \le \; 0$. Since $x_{k + 1}$ satisfies the first-order optimality condition:

34 $$\begin{aligned} \begin{array}{rcl} -2^{-(k + 1)}H_0 \Vert x_{k + 1} - x_k\Vert B(x_{k + 1} - x_k)&\; :=\;&F'(x_{k + 1}) \; \in \; \partial F(x_{k + 1}), \end{array} \end{aligned}$$

we have:

$$\begin{aligned}&\langle B(x_k - x_{k + 1}), x^{*} - x_{k + 1} \rangle \\&\quad \overset{(34)}{=} \frac{2^{k + 1}}{H_0 \Vert x_k - x_{k + 1}\Vert } \langle F'(x_{k + 1}), x^{*} - x_{k + 1} \rangle \; \le \; 0, \end{aligned}$$

where the last inequality follows from the convexity of the objective. $\square$

## Complexity Results for Uniformly Convex Functions

In this section, we are going to justify the global linear rate of convergence of Algorithm 1 for a class of twice differentiable uniformly convex functions with Hölder continuous Hessian. Universality of this method is ensured by the adaptive estimation of the parameter *H* over the whole sequence of iterations. It is important to distinguish two cases: $H_{k + 1} < H_k$ and $H_{k + 1} \ge H_{k}$.

First, we need to estimate the progress in the objective function after minimizing the cubic model. There are two different situations here:

$$\begin{aligned} \hbox {either }H r^{1 - \nu }_H(x) \le \frac{2 {\mathcal {H}}_{\!f}(\nu )}{1 + \nu }, \hbox { or } H r^{1 - \nu }_H(x) > \frac{2 {\mathcal {H}}_{\!f}(\nu )}{1 + \nu }. \end{aligned}$$

### Lemma 4.1

Let $0< {\mathcal {H}}_{\!f}(\nu )< +\infty$ and $\sigma _{\!f}(2 + \nu )> 0$ for some $\nu \in [0,1]$. Then, for arbitrary $x \in {\text {dom}}F$ and $H > 0$ we have:

35 $$\begin{aligned}&F(x) - M_H^{*}(x) \nonumber \\&\quad \ge \; \min \Bigl [ \left( F(x) - F^{*} \right) \cdot \frac{(1 + \nu )}{(2 + \nu )} \cdot \min \bigl \{\bigl ( \frac{(1 + \nu ) \gamma _{f}(\nu )}{2(2 + \nu )} \bigr )^{\frac{1}{1 + \nu }}, \; 1\bigr \} , \nonumber \\&\qquad \left( F(T_H(x)) - F^{*} \right) ^{\frac{3(1 + \nu )}{2(2 + \nu )}} \cdot \bigl ( \frac{2 + \nu }{1 + \nu } \bigr )^{\frac{3(1 + \nu )}{2(2 + \nu )}} \cdot \frac{(\sigma _{\!f}(2 + \nu ))^{\frac{3}{2(2 + \nu )}}}{ 3 \sqrt{H} } \; \Bigr ]. \end{aligned}$$

### Proof

Let us consider two cases. 1) $H r_H^{1 - \nu }(x) \le \frac{2 {\mathcal {H}}_{\!f}(\nu )}{1 + \nu }$. Then, for arbitrary $y \in {\text {dom}}F$, we have:

$$\begin{aligned}&M_H^{*}(x) := Q(x; T_H(x)) + \frac{H}{6}\Vert T_H(x) - x\Vert ^3 + h(T_H(x)) \\&\quad \le Q(x; y) + \frac{H r_H^{1 - \nu }(x) \Vert y - x\Vert ^{2 + \nu }}{2 (2 + \nu ) } + h(y) \\&\quad \overset{(10)}{\le } F(y) + \frac{{\mathcal {H}}_{\!f}(\nu )\Vert y - x\Vert ^{2 + \nu }}{(1 + \nu )(2 + \nu )} + \frac{H r_H^{1 - \nu }(x) \Vert y - x\Vert ^{2 + \nu }}{2 (2 + \nu ) }\\&\quad \le F(y) + \frac{2{\mathcal {H}}_{\!f}(\nu )\Vert y - x\Vert ^{2 + \nu }}{(1 + \nu )(2 + \nu )}, \end{aligned}$$

where the first inequality follows from the fact, that

$$\begin{aligned} T_H(x)= & {} \mathop {\mathrm{argmin}}\limits _{y \in {\text {dom}}F} \bigl \{ Q(x; y) + \frac{H r_H^{1 - \nu }(x) \Vert y - x\Vert ^{2 + \nu }}{2 (2 + \nu ) } + h(y) \bigr \}. \end{aligned}$$

Let us restrict *y* to the segment: $y = \alpha x^{*} + (1 - \alpha ) x,$ with $\alpha \in [0, 1]$. Taking into account the uniform convexity, we get:

$$\begin{aligned}&M_H^{*}(x) \le F(x) - \alpha \left( F(x) - F^{*} \right) + \alpha ^{2 + \nu } \frac{2{\mathcal {H}}_{\!f}(\nu )\Vert x^{*} - x\Vert ^{2 + \nu }}{(1 + \nu )(2 + \nu )} \quad \\&\quad \overset{(4)}{\le } F(x) - \Bigl (\alpha - \alpha ^{2 + \nu } \frac{2{\mathcal {H}}_{\!f}(\nu )}{ (1 + \nu ) \sigma _{\!f}(2 + \nu )} \Bigr ) \left( F(x) - F^{*} \right) . \end{aligned}$$

The minimum of the right-hand side is attained at $\alpha ^{*} = \min \bigl \{\frac{(1 + \nu )\gamma _{f}(\nu )}{2(2 + \nu )}, 1 \bigr \}^{\frac{1}{1 + \nu }}.$ Plugging this value into the bound, we have:

$$\begin{aligned} M^{*}_H(x)\le & {} F(x) - \min \bigl \{ \bigl ( \frac{(1 + \nu )\gamma _{f}(\nu )}{2(2 + \nu )} \bigr )^{1 / (1 + \nu )} , \; 1 \bigr \}\\&\cdot \frac{(1 + \nu )}{(2 + \nu )} \cdot \left( F(x) - F^{*} \right) , \end{aligned}$$

and this is the first argument of the minimum in (35).

2) $H r_H^{1 - \nu }(x) > \frac{2 {\mathcal {H}}_{\!f}(\nu )}{1 + \nu }.$ By (31), we have the bound:

36 $$\begin{aligned} \Vert F'(T_H(x)) \Vert _{*} \; < \; Hr_H^2(x). \end{aligned}$$

Using the fact that $\nabla ^2 f(x) \succeq 0$, we get the second argument of the minimum:

$$\begin{aligned}&F(x) - M_H^{*}(x) \overset{(30)}{\ge } \frac{Hr^3_H(x)}{3} \quad \overset{(36)}{\ge } \quad \frac{\Vert F'(T_H(x)) \Vert _{*}^{\frac{3}{2}}}{3 \sqrt{H}}\\&\quad \quad \overset{(5)}{\ge } \left( \frac{2 + \nu }{1 + \nu } \right) ^{\frac{3(1 + \nu )}{2(2 + \nu )}} \cdot \frac{(\sigma _{\!f}(2 + \nu ))^{\frac{3}{2(2 + \nu )}}}{ 3 \sqrt{H} } \cdot \left( F(T_H(x)) - F^{*} \right) ^{\frac{3(1 + \nu )}{2(2 + \nu )}}. \end{aligned}$$

$$\square $$

Denote by $\kappa _f(\nu )$ the following auxiliary value:

37 $$\begin{aligned} \kappa _f(\nu ) \;:= & {} \; \frac{ {\mathcal {H}}_{\!f}(\nu )^{\frac{2}{1 + \nu }} }{ (\sigma _{\!f}(2 + \nu ))^{\frac{1 - \nu }{(1 + \nu )(2 + \nu )}} } \nonumber \\&\cdot \frac{6 \cdot (8 + \nu )^{\frac{1 - \nu }{1 + \nu }} }{ \left( (1 + \nu )(2 + \nu )\right) ^{\frac{2}{1 + \nu }} } \cdot \bigl ( \frac{1 + \nu }{2 + \nu } \bigr )^{\frac{1 - \nu }{2 + \nu }}, \; \nu \in [0, 1]. \end{aligned}$$

The next lemma shows what happens when parameter *H* is increasing during the iterations.

### Lemma 4.2

Assume that for a fixed $x \in {\text {dom}}F$ the parameter $H > 0$ is such that:

38 $$\begin{aligned} \begin{array}{rcl} F(T_H(x))&\; > \;&M^{*}_H(x). \end{array} \end{aligned}$$

If for some $\nu \in [0, 1]$, we have $\sigma _{\!f}(2 + \nu )> 0$, then it holds:

39 $$\begin{aligned} \begin{array}{rcl} H \left( F(T_{2H}(x)) - F^{*} \right) ^{\frac{1 - \nu }{2 + \nu }} \; < \; \kappa _f(\nu ). \end{array} \end{aligned}$$

### Proof

Firstly, let us prove that from (38) we have:

40 $$\begin{aligned} \begin{array}{rcl} H r^{1 - \nu }_H(x)&\; < \;&\frac{6 {\mathcal {H}}_{\!f}(\nu )}{(1 + \nu )(2 + \nu )}. \end{array} \end{aligned}$$

Assuming by contradiction, $H r^{1 - \nu }_H(x) \; \ge \; \frac{6 {\mathcal {H}}_{\!f}(\nu )}{(1 + \nu )(2 + \nu )}$, we get:

$$\begin{aligned}&M_H^{*}(x) := \frac{H \Vert T_H(x) - x\Vert ^3}{6} + Q(x; T_H(x)) + h(T_H(x)) \\&\quad \quad \ge \frac{{\mathcal {H}}_{\!f}(\nu )\Vert T_H(x) - x\Vert ^{2 + \nu }}{(1 + \nu )(2 + \nu )} + Q(x; T_H(x)) + h(T_H(x)) \\&\quad \quad \overset{(10)}{\ge } F(T_H(x)), \end{aligned}$$

which contradicts (38). Secondly, by its definition, $M^*_H(x)$ is a concave function of *H*. Therefore, its derivative ${d \over d H} M^*_H(x) = {1 \over 6} r_H^3(x)$ is non-increasing. Hence, it holds:

41 $$\begin{aligned} \begin{array}{rcl} r_{2H}(x)\le & {} r_H(x) \overset{(40)}{<} \bigl ( \frac{6 {\mathcal {H}}_{\!f}(\nu )}{(1 + \nu )(2 + \nu ) H} \bigr )^{\frac{1}{1 - \nu }}. \end{array} \end{aligned}$$

Finally, by the smoothness and the uniform convexity, we obtain:

$$\begin{aligned}&H \left( F(T_{2H}(x)) - F^{*} \right) ^{\frac{1 - \nu }{2 + \nu }} \;\; {\mathop {\le }\limits ^{(5)}} \;\; H \left( \frac{1 + \nu }{2 + \nu } \bigl ( \frac{1}{\sigma _{\!f}(2 + \nu )} \bigr )^{\frac{1}{1 + \nu }} \right) ^{\frac{1 - \nu }{2 + \nu }} \Vert F'(T_{2H}(x)) \Vert _{*}^{\frac{1 - \nu }{1 + \nu }} \\&\quad {\mathop {\le }\limits ^{(31)}} H \left( \frac{1 + \nu }{2 + \nu } \bigl ( \frac{1}{\sigma _{\!f}(2 + \nu )} \bigr )^{\frac{1}{1 + \nu }} \right) ^{\frac{1 - \nu }{2 + \nu }} \Bigl ( r^{1 +\nu }_{2H}(x) \cdot \left( \frac{{\mathcal {H}}_{\!f}(\nu )}{1 + \nu } + H r^{1 - \nu }_{2H}(x) \right) \Bigr )^{\frac{1 - \nu }{1 + \nu }} \\&\quad \overset{(41)}{<} H \left( \frac{1 + \nu }{2 + \nu } \bigl ( \frac{1}{\sigma _{\!f}(2 + \nu )} \bigr )^{\frac{1}{1 + \nu }} \right) ^{\frac{1 - \nu }{2 + \nu }} \Bigl ( r^{1+\nu }_{2H}(x) \cdot \frac{(8 + \nu ) {\mathcal {H}}_{\!f}(\nu )}{(1 + \nu )(2 + \nu )} \Bigr )^{\frac{1 - \nu }{1 + \nu }} \\&\quad \overset{(41)}{<} \left( \frac{1 + \nu }{2 + \nu } \bigl ( \frac{1}{\sigma _{\!f}(2 + \nu )} \bigr )^{\frac{1}{1 + \nu }} \right) ^{\frac{1 - \nu }{2 + \nu }} \left( \frac{{\mathcal {H}}_{\!f}(\nu )}{(1 + \nu )(2 + \nu )} \right) ^{\frac{2}{1 + \nu }} 6 (8 + \nu )^{\frac{1 - \nu }{1 + \nu }} \; =: \; \kappa _f(\nu ). \end{aligned}$$

$$\square $$

We are ready to prove the main result of this paper.

### Theorem 4.1

Assume that for a fixed $\nu \in [0, 1]$ we have $0< {\mathcal {H}}_{\!f}(\nu )< +\infty$ and $\sigma _{\!f}(2 + \nu )> 0$. Let parameter $H_0$ in Algorithm 1 be small enough:

42 $$\begin{aligned} \begin{array}{rcl} H_0&\; \le \;&\frac{ \kappa _f(\nu ) }{ \left( F(x_0) - F^{*} \right) ^{(1 - \nu ) / (2 + \nu ) } }, \end{array} \end{aligned}$$

where $\kappa _f(\nu )$ is defined by (37). Let the sequence $\{ x_k \}_{k = 0}^K$ generated by the method satisfy condition:

43 $$\begin{aligned} F(T_{H_k2^{j}}(x_k)) - F^{*} \; \ge \; \varepsilon \; > \; 0, \qquad \quad 0 \le j \le i_k, \quad 0 \le k \le K - 1. \end{aligned}$$

Then, for every $0 \le k \le K - 1$, we have:

44 $$\begin{aligned}&F(x_{k + 1}) - F^{*} \nonumber \\&\quad \le \bigl ( 1 - \min \bigl \{ \frac{ (2 + \nu )\left( (1 + \nu ) (2 + \nu ) \right) ^{ 1 / (1 + \nu ) } \left( \gamma _{f}(\nu )\right) ^{\frac{1}{1 + \nu }} }{ (1 + \nu )6^{3/2} \cdot 2^{1/2} \cdot (8 + \nu )^{ (1 - \nu ) / (2 + 2 \nu ) } } , \frac{1}{2} \bigr \} \bigr ) \cdot \left( F(x_{k}) - F^{*} \right) .\nonumber \\ \end{aligned}$$

Therefore, the rate of convergence is linear, and

$$\begin{aligned} \begin{array}{rcl} K&\; \le \;&\max \bigl \{ \left( \gamma _{f}(\nu )\right) ^{\frac{-1}{1 + \nu }} \cdot \frac{1 + \nu }{2 + \nu } \cdot \frac{6^{3/2} \cdot 2^{1/2} \cdot (8 + \nu )^{(1 - \nu ) / (2 + 2\nu )} }{ \left( (1 + \nu )(2 + \nu ) \right) ^{1 / (1 + \nu )} } , \; 1 \bigr \} \cdot \log \frac{ F(x_0) - F^{*} }{\varepsilon }. \end{array} \end{aligned}$$

Moreover, we have the following bound for the total number of oracle calls $N_K$ during the first *K* iterations:

45 $$\begin{aligned} \begin{array}{rcl} N_K&\; \le \;&2K + \log _2 \frac{\kappa _f(\nu )}{\varepsilon ^{(1 - \nu ) / (2 + \nu )}} - \log _2 H_0. \end{array} \end{aligned}$$

### Proof

The proof is based on Lemmas 4.1 and 4.2, and monotonicity of the sequence $\bigl \{ F(x_k) \bigr \}_{k \ge 0}$. Firstly, we need to show that every iteration of the method is well-defined. Namely, we are going to verify that for a fixed $0 \le k \le K-1$, there exists a finite integer $\ell \ge 0$ such that either $F(T_{H_k 2^{\ell }}(x_k) ) \le M_{H_k 2^{\ell }}^{*}(x_k)$ or $F(T_{H_k 2^{\ell + 1}}(x_k)) - F^{*} < \varepsilon$. Indeed, let us set

46 $$\begin{aligned} \begin{array}{rcl} \ell := \max \left\{ 0, \log _2 \left\lceil \frac{\kappa _f(\nu )}{H_k \varepsilon ^{(1 - \nu ) / (2 + \nu )} } \right\rceil \right\} ,&\quad \text {and} \quad&H := H_k 2^{\ell } \ge \frac{\kappa _f(\nu )}{\varepsilon ^{(1 - \nu ) / (2 + \nu )}}. \end{array} \end{aligned}$$

Then, if we have both $F(T_{H}(x_k)) > M_{H}^{*}(x_k)$ and $F(T_{2H}(x_k)) - F^{*} \ge \varepsilon$, we get by Lemma 4.2:

$$\begin{aligned} \begin{array}{rcl} H&\; \overset{(39)}{<} \;&\frac{\kappa _f(\nu )}{\left( F(T_{2H}(x_k)) - F^{*} \right) ^{(1 - \nu ) / (2 + \nu )} } \; \le \; \frac{\kappa _f(\nu )}{\varepsilon ^{(1 - \nu ) / (2 + \nu )}}, \end{array} \end{aligned}$$

which contradicts (46). Therefore, if we are unable to find the value $0 \le i_k \le \ell$ (see line 1 of Algorithm) in a finite number of steps, that only means we have already solved the problem up to accuracy $\varepsilon$.

Now, let us show that for every $0 \le k \le K$ it holds:

47 $$\begin{aligned} \begin{array}{rcl} H_k \left( F(x_k) - F^{*} \right) ^{\frac{1 - \nu }{2 + \nu }}\le & {} \max \left\{ \kappa _f(\nu ), \; H_0 \left( F(x_0) - F^{*} \right) ^{\frac{1 - \nu }{2 + \nu }} \right\} . \end{array} \end{aligned}$$

This inequality is obviously valid for $k = 0$. Assume it is also valid for some $k \ge 0$. Then, by definition of $H_{k + 1}$ (see line 3 of Algorithm), we have $H_{k + 1} = H_k 2^{i_k - 1}$. There are two cases. 1) $i_k = 0$. Then, $H_{k + 1} < H_k$. By monotonicity of $\bigl \{ F(x_k) \bigr \}_{k \ge 0}$ and by induction, we get:

$$\begin{aligned} \begin{array}{rcl} H_{k + 1} \left( F(x_{k + 1}) - F^{*} \right) ^{\frac{1 - \nu }{2 + \nu }} &{} \; < \; &{} H_k \left( F(x_k) - F^{*} \right) ^{\frac{1 - \nu }{2 + \nu }} \\ &{} \; \le \; &{} \max \left\{ \kappa _f(\nu ), \; H_0 \left( F(x_0) - F^{*} \right) ^{\frac{1 - \nu }{2 + \nu }} \right\} . \end{array} \end{aligned}$$

2) $i_k > 0$. Then, applying Lemma 4.2 with $H := H_k 2^{i_k - 1} = H_{k + 1}$ and $x := x_{k}$, we have:

$$\begin{aligned} \begin{array}{rcl} H_{k + 1} \left( F(x_{k + 1}) - F^{*} \right) ^{\frac{1 - \nu }{2 + \nu }} \; = \; H \left( F(T_{2H}(x)) - F^{*} \right) ^{\frac{1 - \nu }{2 + \nu }} \; \overset{(39)}{\le } \; \kappa _f(\nu ). \end{array} \end{aligned}$$

Thus, (47) is true by induction. Choosing $H_0$ small enough (42), we have:

48 $$\begin{aligned} \begin{array}{rcl} 2H_{k} \left( F(x_k) - F^{*}\right) ^{\frac{1 - \nu }{2 + \nu }}&\; \le \;&2\kappa _f(\nu ), \qquad 0 \le k \le K. \end{array} \end{aligned}$$

From Lemma 4.1 we know, that one of the two following estimates is true (denote $\delta _k := F(x_k) - F^{*}$):

1. $$F(x_k) - F(x_{k + 1}) \ge \alpha \cdot \delta _k \; \Leftrightarrow \; \delta _{k + 1} \le (1 - \alpha ) \cdot \delta _k, \; $$ *or*
2. $$F(x_k) - F(x_{k + 1}) \ge \beta \cdot \delta _{k + 1} \; \Leftrightarrow \; \delta _{k + 1} \le (1 + \beta )^{-1} \delta _k \le (1 - \min \{\beta , 1\} / 2) \cdot \delta _k$$,

where $$ \alpha := \frac{1 + \nu }{2 + \nu } \cdot \min \bigl \{\bigl ( \frac{(1 + \nu ) \gamma _{f}(\nu )}{2(2 + \nu )} \bigr )^{ \frac{1}{1 + \nu }}, \; 1\bigr \}, $$ and

$$\begin{aligned} \begin{array}{rcl} \beta:= & {} \bigl ( \frac{2 + \nu }{1 + \nu } \bigr )^{\frac{3(1 + \nu )}{2(2 + \nu )}} \cdot \frac{(\sigma _{\!f}(2 + \nu ))^{\frac{3}{2(2 + \nu )}}}{3 (2 \kappa _f(\nu ))^{1/2} } \; {\mathop {=}\limits ^{(37)}} \; \frac{2 + \nu }{1 + \nu } \cdot \frac{ 2^{1/2} \cdot \left( (1 + \nu ) (2 + \nu ) \right) ^{\frac{1}{1 + \nu }} }{6^{3/2} \cdot (8 + \nu )^{(1 - \nu ) / (2 + 2\nu )}} \cdot \gamma _{f}(\nu )^{\frac{1}{1 + \nu }}. \end{array} \end{aligned}$$

It remains to notice that $\alpha \ge \min \bigl \{\beta , 1\bigr \} / 2$. Thus, we obtain (44).

Finally, let us estimate the total number of the oracle calls $N_K$ during the first *K* iterations. At each iteration, the oracle is called $i_k + 1$ times, and we have $H_{k + 1} = H_k 2^{i_k - 1}$. Therefore,

$$\begin{aligned} N_K= & {} \sum _{k = 0}^{K - 1} (i_k + 1) \; = \; \sum _{k = 0}^{K - 1} \left( \log _2 \frac{H_{k + 1}}{H_k} + 2\right) \\= & {} 2K + \log _2 H_{K} - \log _2 H_0 \; \overset{(48), (43)}{\le } \; 2K + \log _2 \frac{\kappa _f(\nu )}{\varepsilon ^{(1 - \nu ) / (2 + \nu )}} - \log _2 H_0. \end{aligned}$$

$$\square $$

Note that condition (42) for the initial choice of $H_0$ can be seen as a definition of the moment, after which we can guarantee the linear rate of convergence (44). In practice, we can launch Algorithm 1 with *arbitrary* $H_0 > 0$. There are two possible options: either the method halves $H_k$ at every step in the beginning, so $H_k$ becomes small very quickly, or this value is increased at least once, and the required bound is guaranteed by Lemma 4.2. It can be easily proved, that this initial phase requires no more than $K_0 = \bigl \lceil \log _2 \frac{H_0 \varepsilon ^{(1 - \nu ) / (1 + \nu )}}{\kappa _f(\nu )} \bigr \rceil$ oracle calls.

## Discussion

Let us discuss the global complexity results, provided by Theorem 4.1 for the Cubic Regularization of the Newton Method with the adaptive adjustment of the regularization parameter.

For the class of twice continuously differentiable strongly convex functions with Lipschitz continuous gradients $f \in {\mathcal {S}}_{\mu , L}^{2, 1}({\text {dom}}F)$, it is well known that the classical gradient descent method needs

49 $$\begin{aligned} \begin{array}{c} O\bigl ( \frac{L}{\mu } \log \frac{F(x_0) - F^{*}}{\varepsilon } \bigr ) \end{array} \end{aligned}$$

iterations for computing $\varepsilon$-solution of the problem (e.g., [15]). As it was shown in [6], this result is shared by a variant of Cubic Regularization of the Newton method. This is much better than the bound $O\bigl ( \bigl (\frac{L}{\mu }\bigr )^2 \log \frac{F(x_0) - F^{*}}{\varepsilon }\bigr ),$ known for the damped Newton method (e.g., [2]).

For the class of uniformly convex functions of degree $p = 2 + \nu$ having Hölder continuous Hessian of degree $\nu \in [0, 1]$, we have proved the following parametric estimates: $O\bigl ( \max \bigl \{ \bigl (\gamma _{f}(\nu )\bigr )^{\frac{-1}{1 + \nu }}, 1\bigr \} \cdot \log \frac{F(x_0) - F^{*}}{\varepsilon } \bigr ),$ where $\gamma _{f}(\nu ):=\frac{\sigma _{\!f}(2 + \nu )}{{\mathcal {H}}_{\!f}(\nu )}$ is the *condition number* of degree $\nu$. However, in practice we may not know exactly an appropriate value of the parameter $\nu$. It is important that our algorithm automatically adjusts to the best possible complexity bound:

50 $$\begin{aligned} \begin{array}{c} O\bigl ( \max \bigl \{ \; \inf _{\nu \in [0, 1]} \bigl (\gamma _{f}(\nu )\bigr )^{\frac{-1}{1 + \nu }}, \; 1 \;\bigr \} \cdot \log \frac{F(x_0) - F^{*}}{\varepsilon } \bigr ). \end{array} \end{aligned}$$

Note that for $f \in S_{\mu , L}^{2, 1}({\text {dom}}F)$ we have:

$$\begin{aligned} \begin{array}{rcl} \Vert \nabla ^2 f(x) - \nabla ^2 f(y) \Vert \; \le \; L - \mu , \qquad x, y \in {\text {dom}}F. \end{array} \end{aligned}$$

Thus, ${\mathcal {H}}_{\!f}(0) \le L-\mu$ and $\gamma _f(0) \ge \frac{\mu }{L - \mu }$. So we can conclude that the estimate (50) is better than (49). Moreover, addition to our objective *arbitrary* convex quadratic function does not change any of ${\mathcal {H}}_{\!f}(\nu ), \, \nu \in [0, 1]$. Thus, it can only improve the condition number $\gamma _{f}(\nu )$, while the ratio $L / \mu$ may become arbitrarily bad. It confirms an intuition that a natural Newton-type minimization scheme should not be affected by any quadratic parts of the objective, and the notion of *well-conditioned* and *ill-conditioned* problems for second-order methods should be different from that of for first-order ones.

Note that in the recent paper [11], a linear rate of convergence was also proven for the accelerated second-order scheme, with the complexity bound:

51 $$\begin{aligned} \begin{array}{c} O\bigl ( \max \{ (\gamma _{f}(\nu ))^{\frac{-1}{2 + \nu }}, 1\} \cdot \log \frac{{\mathcal {H}}_{\!f}(\nu )D_0^{2 + \nu }}{\varepsilon } \bigr ). \end{array} \end{aligned}$$

This is the better rate than (50). However, the method requires to know the parameter $\nu$, and the constant of uniform convexity. Thus, one theoretical question remains open: is it possible to construct *universal* second-order scheme, matching (51) in the uniformly convex case.

Looking at the definitions of ${\mathcal {H}}_{\!f}(\nu )$ and $\sigma _{\!f}(2 + \nu )$, we can see that, for all $x, y \in {\text {dom}}F, x \not = y$,

$$\begin{aligned} \begin{array}{rcccl} \sigma _{\!f}(2 + \nu )\; \le \; \frac{\langle \nabla f(x) - \nabla f(y), x - y\rangle }{\Vert x - y\Vert ^{2 + \nu }}, \quad \frac{1}{{\mathcal {H}}_{\!f}(\nu )} \; \le \; \frac{\Vert x - y\Vert ^{\nu }}{\Vert \nabla ^2 f(x) - \nabla ^2 f(y) \Vert }, \end{array} \end{aligned}$$

and

$$\begin{aligned} \begin{array}{rcl} \gamma _{f}(\nu )\; := \; \frac{\sigma _{\!f}(2 + \nu )}{{\mathcal {H}}_{\!f}(\nu )} \; \le \; \frac{\langle \nabla f(x) - \nabla f(y), x - y \rangle }{\Vert \nabla ^2 f(x) - \nabla ^2 f(y)\Vert \cdot \Vert x - y\Vert ^2}. \end{array} \end{aligned}$$

The last fraction does not depend on any particular $\nu$. So, for any twice-differentiable convex function, we can define the following number:

$$\begin{aligned} \begin{array}{rcl} \gamma _f :=\inf \limits _{\begin{array}{c} x, y \in {\text {dom}}F \\ x \not = y \end{array}} \frac{\langle \nabla f(x) - \nabla f(y), x - y \rangle }{\Vert \nabla ^2 f(x) - \nabla ^2 f(y) \Vert \cdot \Vert x - y\Vert ^2}. \end{array} \end{aligned}$$

If it is positive, then it could serve as an indicator of the *second-order non-degeneracy*, for which we have a lower bound: $\gamma _f \ge \gamma _{f}(\nu ), \; \nu \in [0, 1].$

## Conclusions

In this work, we have introduced the second-order condition number of a certain degree, which plays as the main complexity factor for solving uniformly convex minimization problems with Hölder-continuous Hessian of the objective by second-order optimization schemes.

We have proved that cubically regularized Newton method with an adaptive estimation of the regularization parameter achieves global linear rate of convergence on this class of functions. The algorithm does not require to know any parameters of the problem class and automatically fits to the best possible degree of nondegeneracy.

Using this technique, we have justified that global iteration complexity of cubic Newton is always better than corresponding one of gradient method for the standard class of strongly convex functions with uniformly bounded second derivative.

Data sharing not applicable to this article as no datasets were generated or analyzed during the current study.

## Footnotes

1 $$^{)}$$ For the integer values of *p*, this inequality was proved in [14].
